# Increased Flavonol Levels in Tobacco Expressing *AcFLS* Affect Flower Color and Root Growth

**DOI:** 10.3390/ijms21031011

**Published:** 2020-02-04

**Authors:** Sangkyu Park, Da-Hye Kim, Ju-Hee Yang, Jong-Yeol Lee, Sun-Hyung Lim

**Affiliations:** National Institute of Agricultural Sciences, Rural Development Administration, JeonJu 54874, Korea; psk2779@korea.kr (S.P.); kimdh143@jbnu.ac.kr (D.-H.K.); didwngml77@naver.com (J.-H.Y.); jy0820@korea.kr (J.-Y.L.)

**Keywords:** *Allium cepa*, anthocyanin, flavonoid, flavonol, flavonol synthase, flower color, root growth

## Abstract

The onion (*Allium cepa* L.) *flavonol synthase* (*AcFLS-HRB*) gene, encoding an enzyme responsible for flavonol biosynthesis in yellow onion, was recently identified and enzymatically characterized. Here, we performed an in vivo feeding assay involving bacterial expression of AcFLS-HRB and observed that it exhibited both flavanone 3-hydroxylase (F3H) and FLS activity. Transgenic tobacco (*Nicotiana tabacum*) expressing *AcFLS-HRB* produced lighter-pink flowers compared to wild-type plants. In transgenic petals, *AcFLS-HRB* was highly expressed at the mRNA and protein levels, and most AcFLS-HRB protein accumulated in the insoluble microsomal fractions. High-performance liquid chromatography (HPLC) analysis showed that flavonol levels increased but anthocyanin levels decreased in transgenic petals, indicating that *AcFLS-HRB* is a functional gene in planta. Gene expression analysis showed the reduced transcript levels of general phenylpropanoid biosynthetic genes and flavonoid biosynthetic genes in *AcFLS-HRB* overexpressed tobacco petals. Additionally, transgenic tobacco plants at the seedling stages showed increased primary root and root hair length and enhanced quercetin signals in roots. Exogenous supplementation with quercetin 3-*O*-rutinoside (rutin) led to the same phenotypic changes in root growth, suggesting that rutin is the causal compound that promotes root growth in tobacco. Therefore, augmenting flavonol levels affects both flower color and root growth in tobacco.

## 1. Introduction

Flavonoids, one of the largest groups of plant secondary metabolites, are involved in plant growth, development, reproduction, and responses to biotic and abiotic stress [1]. More than 10,000 different flavonoid compounds have been described [2,3], which are categorized into six subclasses based on their structural properties: flavanones, flavones, isoflavones, flavonols, proanthocyanidins, and anthocyanins [4]. Flavonols are the most abundant flavonoid compounds in plants and have received much attention due to their various physiological roles, including the regulation of auxin transport [5], symbiont attraction [6], male fertility [7,8], protection against UV-B irradiation [9], and the recruitment of pollinators [10].

**Figure 1:**
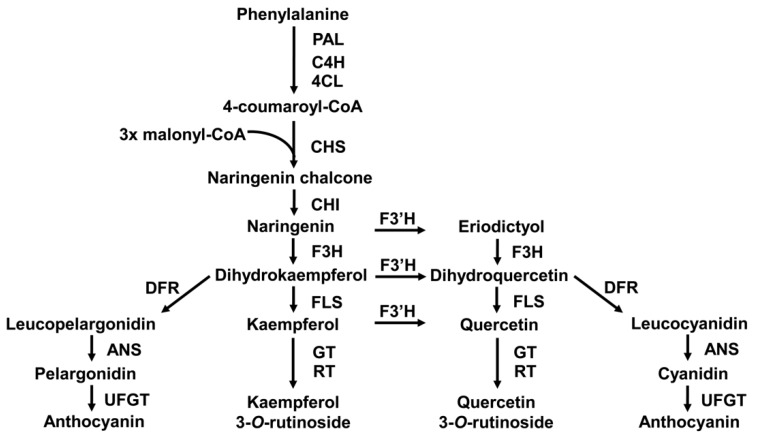
Flavonoid biosynthesis in plants. PAL, phenylalanine ammonia lyase; C4H, cinnamic acid 4-hydroxylase; 4CL, 4-coumaric acid:CoA ligase; CHS, chalcone synthase; CHI, chalcone isomerase; F3ʹH, flavonoid 3ʹ-hydroxylase; F3H, flavanone 3-hydroxylase; FLS, flavonol synthase; DFR, dihydroflavonol 4-reductase; ANS, anthocyanidin synthase; GT, glucosyltransferases; RT, rhamnosyltransferase; UFGT, UDP-glucose:flavonoid-3-*O*-glucosyltransferase.

The flavonoid biosynthesis pathway was thoroughly investigated, and most of the enzymes involved were identified in several plant species [10,11,12,13]. Phenylalanine ammonia lyase (PAL) catalyzes the deamination of phenylalanine to yield cinnamic acid, which is the first committed step in phenylpropanoid biosynthesis. The flavonoid biosynthesis pathway is initiated by the activity of chalcone synthase (CHS), an important regulatory point that affects the metabolic flux of flavonoids, which condenses one molecule of 4-coumaroyl CoA generated through the phenylpropanoid pathway with three molecules of malonyl-CoA, resulting in chalcone formation. Chalcone is further converted to the (2S)-flavanone naringenin by chalcone isomerase (CHI). From this central intermediate naringenin, diverse classes of flavonoids are generated through several side branches. Flavanone 3-hydroxylase (F3H) catalyzes the conversion of (2S)-flavanones (e.g., naringenin and eriodictyol) to dihydroflavonols (e.g., dihydrokaempferol and dihydroquercetin). Flavonol synthase (FLS) and dihydroflavonol 4-reductase (DFR) compete for the dihydroflavanols to produce flavonol aglycones (e.g., kaempferol and quercetin) and leucoanthocyanidins, respectively. Once the flavonol aglycones are biosynthesized, the glycosylation of flavonols is catalyzed by glucosyltransferase (GT) and rhamnosyltransferase (RT), which are active in the cytosol and render hydrophobic flavonols more soluble and less toxic to facilitate their transport and storage [14,15,16,17,18,19]. Leucoanthocyanidins are converted to anthocyanidins (e.g., pelargonidin and cyanidin) by anthocyanidin synthase (ANS) and further glycosylated by UDP-glucose:flavonoid 3-*O*-glucosyltransferase (UFGT), resulting in anthocyanins. Flavonoid 3ʹ-hydroxylase (F3ʹH) catalyzes 3ʹ-hydroxylation of the flavonoid B-ring, which imparts diversity to flavonoid molecules (Figure 1). 

The competition between FLS and DFR for dihydroflavonols creates a critical branch point separating the flavonol and anthocyanin biosynthetic pathways. Several studies used antisense down-regulation or the overexpression of *FLS* and *DFR* to characterize these genes and their roles in regulating flower color [20,21,22]. Antisense *FLS* in the flowers of *Eustoma grandiflorum Grise*, *Petunia hybrida*, and *Mimulus lewisii* caused a decrease in flavonol contents and an increase in anthocyanin levels, resulting in flowers with enhanced coloration. Conversely, antisense *DFR* in tobacco led to an increase in flavonol contents and a decrease in anthocyanin contents, resulting in white flowers [23]. When *FLS* genes from *Rosa rugosa*, *Prunus persica*, or *Petunia hybrida* were expressed in tobacco, the resulting flowers contained increased levels of flavonol and decreased levels of anthocyanin, while transgenic tobacco expressing *DFR* genes from *Rosa rugosa* or *Petunia hybrida* showed the opposite phenotypes [24]. These inverse correlations between flavonol and anthocyanin production are commonly observed. Several transcription factor families, including R2R3-MYB, basic helix–loop–helix (bHLH), WD40, are involved in the transcriptional control of phenylpropanoid and flavonoid biosynthesis genes [25,26,27,28]. Among these, certain R2R3-MYB and bHLH transcription factors are involved in regulating *FLS* or *DFR* expression. The heterologous expression of the *Arabidopsis thaliana* gene *MYB12* in tobacco enhanced the expression of *FLS* and resulted in the accumulation of flavonols, demonstrating that AtMYB12 positively regulates *FLS* [29]. In *Zea mays*, *ZmFLS1* is directly up-regulated by the collaborative activity of the R2R3-MYB transcription factor C1 and bHLH transcription factor R [30], while in *Malus × domestica*, MYB10 positively regulates *DFR* expression and therefore anthocyanin accumulation [31]. However, the mechanism explaining the inverse correlation between flavonol and anthocyanin biosynthesis has not yet been clarified.

Flavonols are important regulators not only of flower color, but also of root growth and development. Flavonols inhibit polar auxin transport (PAT), a process mediated by several auxin transporters [32]. The intercellular migration of auxin is closely related to the asymmetric subcellular localization of the major auxin efflux carriers PINs, which can be altered by phosphorylation of PINs by protein kinase PINOID [33]. Flavonols inhibit PINOID activity, thus reducing the phosphorylation level of PIN2, leading to a change in PIN2 localization. Therefore, flavonols play important roles in determining whether auxin flow is directed towards the shoot or root [33]. The reactive oxygen species (ROS)-scavenging activity of flavonols highlights their importance in root growth and development. Auxin triggers the production of O_2_^−^, which promotes cell division in the meristematic zone, whereas cytokinin and H_2_O_2_ cooperatively arrest cell division and initiate differentiation in the elongation and differentiation zones [34,35]. This antagonistic relationship between auxin-O_2_^−^ and cytokinin-H_2_O_2_ controls the balance between cell division and differentiation in the root. Cytokinin and H_2_O_2_ induce flavonol biosynthesis, which inhibits root-ward PAT by disturbing PIN localization and scavenging O_2_^−^ to reduce cell division in the meristem [35]. Therefore, in *Arabidopsis*, increased flavonol levels result in reduced primary root length, whereas the suppression of flavonol accumulation causes the opposite phenotype [36]. However, the tomato mutant *anthocyanin reduced* (*are*), harboring deficient *F3H* and significantly reduced flavonol levels, has shorter primary roots than the wild-type [37]. These findings indicate that the patterns of flavonol-mediated root growth differ depending on the plant species, since root phenotypes result from the integrated signaling of PAT, ROS, and flavonol. 

Both kaempferol and quercetin are active components of PAT. However, the derivatives of each compound are thought to play different roles in PAT. The accumulation of kaempferol-3-*O*-glucoside in the *rol1-2* mutant (harboring a mutation in *rhamnose synthase 1*) is responsible for the positive regulation of PAT in the shoot and its negative regulation in the root [38]. The accumulation of kaempferol 3-*O*-rhamnoside-7-*O*-rhamnoside in the *ugt78d2* mutant (harboring a mutation in the *flavonoid 3-O-glucosyltransferase* gene) is responsible for the negative regulation of PAT in the shoot [39]. Finally, the accumulation of quercetin-3-*O*-rhamnoside in *WRKY23* overexpression (activator of *F3ʹH*) plants is responsible for the negative regulation of PAT in the root [40]. The number of hydroxyl groups on the B-rings of flavonols differentially affects their ability to scavenge free radicals, and other types of modifications, such as glycosylation, methylation, and acylation, cause variations in the antioxidant capacities for different types of ROS [41]. Therefore, the specific flavonol compositions in different plant species may have different effects on regulating root growth and development.

Several *FLS* genes were identified from dicot and monocot plants, and their enzymatic properties and/or functionalities were characterized in planta [42,43,44,45,46,47,48,49,50]. Among these, AtFLS1, CitFLS, GbFLS, and OsFLS are bifunctional enzymes exhibiting both F3H and FLS activity [42,44,46,50]. Recently, two *FLS* genes (*AcFLS-HRB* and *AcFLS-H6*) were identified in two different colored onion (*Allium cepa*) cultivars, the yellow onion ‘Hwangryongball’ (‘HRB’) and ‘H6′, a double haploid line of red onion, respectively [49]. Enzymatic characterizations of the two *AcFLS* genes revealed that the preferred substrate of both enzymes is dihydroquercetin (DHQ) over dihydrokaempferol (DHK) and that AcFLS-HRB exhibits higher catalytic efficiency than AcFLS-H6. Here, we carried out an in vivo feeding assay via bacterial expression of AcFLS-HRB and found that AcFLS-HRB is a bifunctional enzyme with both F3H and FLS activity. We verified its functions in planta through phenotypic, molecular, and biochemical analysis of transgenic tobacco expressing AcFLS-HRB. Transgenic tobacco produced lighter-pink flowers containing higher flavonol levels and lower anthocyanin levels than the wild-type. In accordance with these phenotypes, phenylpropanoid biosynthesis genes and several flavonoid biosynthetic genes were down-regulated in transgenic petals. We also observed changes in root growth in the transgenic tobacco plants, with longer primary roots and root hairs than the wild-type, which was consistent with the accumulation patterns of quercetins in the roots. These findings indicate that increased quercetin levels promote root growth in tobacco. 

## 2. Results

### 2.1. Recombinant AcFLS-HRB Protein Exhibits both F3H and FLS Activity 

**Figure 2:**
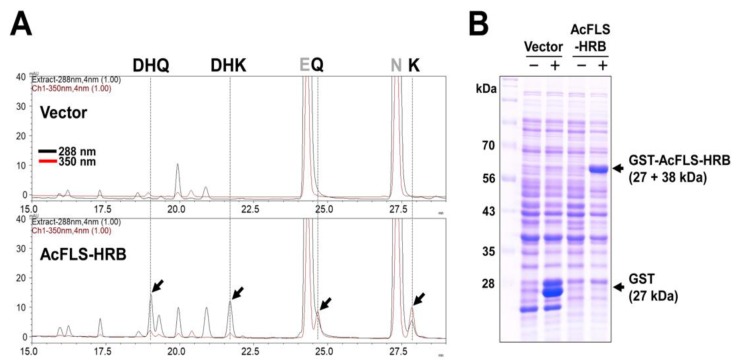
In vivo substrate-feeding assay of AcLFS-HRB enzyme activity. (**A**). *E. coli* strain BL21 cells harboring recombinant GST (Vector) and expressing GST-AcFLS-HRB were simultaneously fed with flavanones (naringenin [N] and eriodictyol [E]), and the resulting dihydroflavonols (DHK and DHQ) and flavonols (kaempferol [K] and quercetin [Q]) were analyzed by HPLC. Flavanones and dihydroflavonols were detected at 288 nm, and flavonols were detected at 350 nm. Arrows indicate products from the bacterial culture expressing AcFLS-HRB. (**B**). Recombinant protein expression induced by IPTG (-, noninduced; +, induced) was confirmed by SDS-PAGE.

To determine whether AcFLS-HRB is a bifunctional enzyme, we expressed recombinant AcFLS-HRB protein fused with GST (glutathione S-transferase) in *E. coli* and measured its activity by substrate-feeding in bacterial culture medium. Equal amounts of racemic flavanone substrates (naringenin and eriodictyol) were simultaneously added to bacterial cultures expressing GST only and GST-AcFLS-HRB after isopropyl β-D-1-thiogalactopyranoside (IPTG) induction. After 3 h of incubation, we extracted the products and analyzed them by high-performance liquid chromatography (HPLC) (Figure 2A). SDS-PAGE confirmed that recombinant proteins were successfully expressed in the cultures (Figure 2B). HPLC analysis showed that both dihydroflavonols (DHK and DHQ) and flavonols (kaempferol and quercetin) were produced in cultures expressing GST-AcFLS-HRB, indicating that GST-AcFLS-HRB converted flavanone substrates to dihydroflavonols and further converted the dihydroflavonols to flavonols. These results indicate that GST-AcFLS-HRB exhibits F3H activity as well as FLS activity. 

### 2.2. Transgenic Tobacco Expressing AcFLS-HRB Has Lighter-Pink Flowers than the Wild-Type 

**Figure 3:**
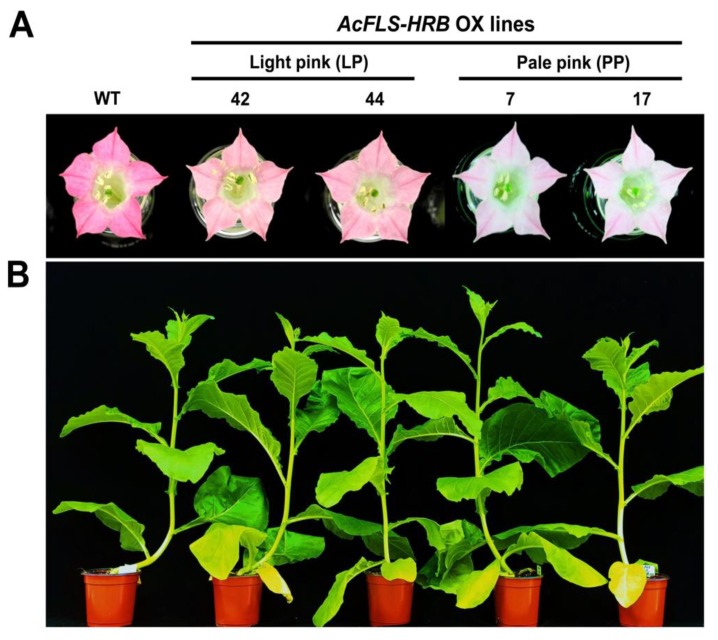
Phenotypes of transgenic tobacco expressing *AcFLS*-*HRB*. Phenotypes of flowers (**A**) and 9-week-old plants (**B**) expressing *AcFLS*-*HRB.* WT, wild-type; 42, 44, 7, and 17, transgenic lines with light pink/pale pink flowers.

We obtained more than 20 T_0_ transgenic tobacco (*Nicotiana tabacum* cv Xanthi) lines expressing *AcFLS-HRB* driven by the CaMV 35S promoter and their seeds were obtained from self-pollinated plants. After progressing to the T_1_ generation, their phenotypes were analyzed. Compared to wild-type (WT) flowers, the flowers of T_1_ transgenic lines showed decreases in pinkness to various extents (Figure 3A). Based on their flower colors, the T_1_ lines were divided into two groups, light pink (LP) and pale pink (PP), and two representative plants were selected from each group. On the other hand, no phenotypic differences in growth and development between WT and the transgenic lines were observed (Figure 3B). 

### 2.3. Expression of AcFLS-HRB in Tobacco Increases Flavonol and Decreases Anthocyanin Levels in Flowers 

**Figure 4:**
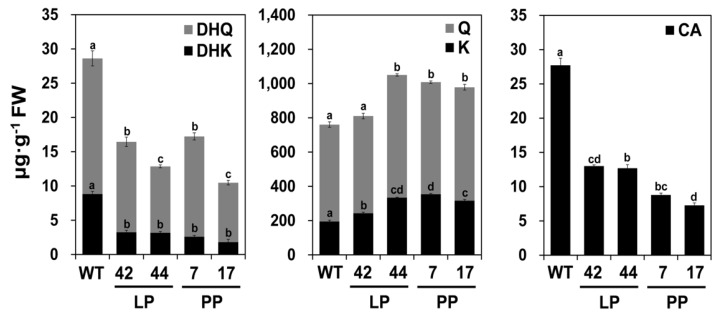
Flavonoid contents in the petals of transgenic tobacco flowers expressing AcFLS-HRB. A, HPLC analysis of flavonoid aglycones extracted from the petals of WT and transgenic lines (42, 44, 7, 17) via acid hydrolysis. Peaks corresponding to dihydroflavonols (DHQ and DHK), flavonols (Q, quercetin; K, kaempferol), and cyanidin (CA) were detected at 288 nm, 350 nm, and 520 nm, respectively. B, Flavonoid contents were measured in accordance with the area of each standard. Error bars indicate ± SD from three replicates. Statistical significance was determined by one-way ANOVA with post-hoc Tukey’s tests. Significant differences between means (*p* < 0.05) are indicated by lower-case letters (a, b, c, and d) above the bars. LP, light pink transgenic lines; PP, pale pink transgenic lines.

Acid-hydrolyzed flavonoid aglycones were extracted from their flower petals and analyzed using HPLC, and their contents were calculated based on the areas of corresponding standards. The dihydroflavonol levels in transgenic petals were approximately 50% those of WT petals (Figure 4). The DHK levels were much more greatly reduced than the DHQ levels, with decreases of 63% to 81% and 25% to 58%, respectively, compared to the WT. By contrast, the levels of flavonols, including kaempferol and quercetin, tended to increase with decreasing intensity of the pink coloration in petals, with overall changes of 5% to 28% in the LP lines and 20% to 24% in the PP lines relative to the WT. The levels of quercetin in the petals were generally higher than those of kaempferol; however, the increase in kaempferol levels in the transgenic plants (17–49%) was more pronounced than the increase in quercetin levels (7–10%, except for line 42). The levels of cyanidin-derived anthocyanins, the major class of anthocyanins in the petals of tobacco flowers, were greatly reduced in transgenic petals relative to the WT, with the largest decreases (71–76%) observed in the PP lines. These results indicate that the functional expression of *AcFLS-HRB* in tobacco caused an increase in flavonol accumulation and a decrease in anthocyanin accumulation in the transgenic petals, resulting in light pink and pale pink flowers.

### 2.4. Anthocyanin Biosynthesis Genes Are Down-Regulated in AcFLS-HRB Transgenic Tobacco Petals 

**Figure 5:**
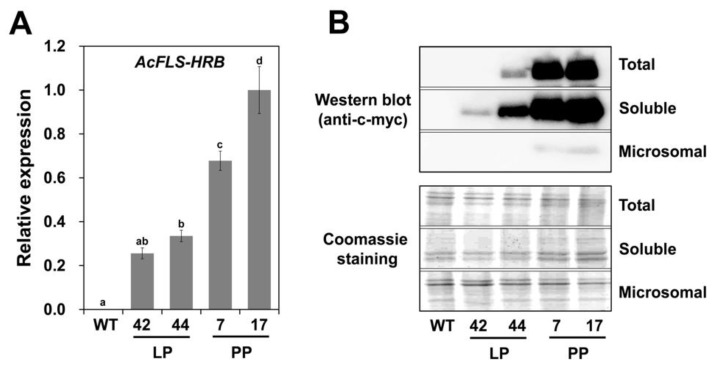
*AcFLS-HRB* expression and protein contents in WT and *AcFLS-HRB-*expressing transgenic tobacco petals. (**A**), Relative expression levels of *AcFLS-HRB* in WT and transgenic tobacco petals, as revealed by qPCR. Error bars indicate ± SD from three replicates. Statistical significance was determined by one-way ANOVA with post-hoc Tukey’s tests. Significant differences between means (*p* < 0.05) are indicated by lower-case letters (a, b, c, and d) above the bars. (**B**), Immuno blot analysis of WT and transgenic petals using anti-c-myc antibody. Coomassie staining was conducted for the loading control. LP, light pink transgenic lines; PP, pale pink transgenic lines.

We examined *AcFLS-HRB* transcript abundance and AcFLS-HRB protein levels in transgenic tobacco petals by quantitative RT-PCR (qPCR) and immunoblot analysis, respectively. *AcFLS-HRB* was highly expressed in transgenic petals, with higher transcript levels in the PP lines vs. the LP lines (Figure 5A). Consistently, AcFLS-HRB protein was only present in the transgenic lines, with very high levels in the PP lines (Figure 5B). Notably, most of the proteins were detected in the soluble fraction of total extracts rather than the insoluble microsomal fraction, indicating that the majority of AcFLS-HRB protein was present in a soluble form in petal cells. 

**Figure 6:**
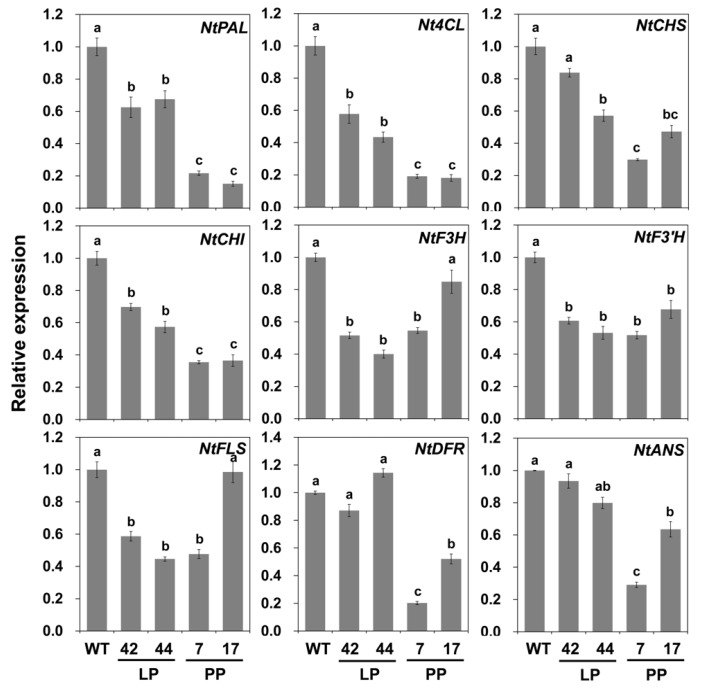
Expression patterns of flavonoid biosynthesis pathway genes in WT and *AcFLS-HRB-*expressing tobacco petals, as revealed by qPCR. LP, light pink transgenic lines; PP, pale pink transgenic lines. Error bars indicate ± SD from three replicates. Statistical significance was determined by one-way ANOVA with post-hoc Tukey’s tests. Significant differences between means (*p* < 0.05) are indicated by lower-case letters (a, b, and c) above the bars.

Unlike *AcFLS-HRB*, the flavonoid biosynthetic genes were generally down-regulated in transgenic petals (Figure 6). Genes of the phenylpropanoid biosynthesis pathway, such as *NtPAL* and *Nt4CL*, and the early biosynthetic genes (EBGs) in the flavonoid pathway, including *NtCHS* and *NtCHI*, were significantly down-regulated. In the case of the late biosynthetic genes (LBGs) involved in anthocyanin biosynthesis, such as *NtDFR* and *NtANS*, there was little to no repression in the LP lines but significant down-regulation in the PP lines, corresponding to the degree of reduction in the pigmentation of the petals. These results indicate that the transgenic expression of *AcFLS-HRB* could affect the metabolic flux of flavonoid biosynthesis and that the higher levels of *AcFLS-HRB* expression in the PP lines could accelerate the decrease in anthocyanin biosynthesis through the suppression of *DFR* and *ANS* expression in tobacco petals. 

### 2.5. Increased Flavonol Levels in Tobacco Expressing AcFLS-HRB Promote Primary Root and Root Hair Growth 

**Figure 7:**
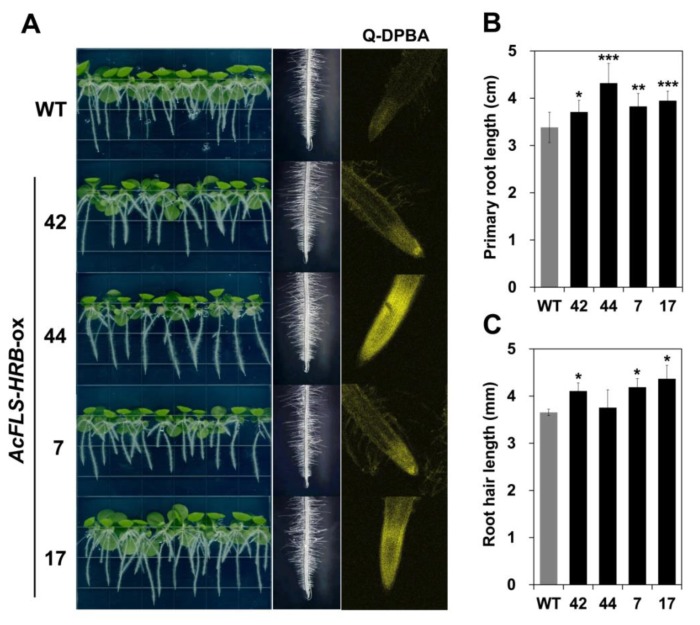
Changes in the root phenotypes of *AcFLS-HRB*-expressing tobacco seedlings. (**A**). Phenotypes of 16-day-old wild-type and transgenic (T_3_) seedlings grown vertically on Murashige and Skoog (MS) medium (without sucrose) (left column). The root hair (middle column) and the DPBA staining of flavonols in the roots (right column) were observed by microscopic examination. (**B**), Average primary root length of 30 seedlings. (**C**), Average root hair length of at least eight primary roots. Data are expressed as mean ± SD, as determined from the replicates. Significant differences were determined by Student’s *t*-test. Asterisks indicate that the value is significantly different from that of the WT (* *p* < 0.05, ** *p* < 0.01, *** *p* < 0.001).

To investigate whether the increased flavonol levels in *AcFLS-HRB*-expressing transgenic tobacco affect seedling growth, we examined T_3_ transgenic seedlings grown on vertical plates. The primary roots and root hairs were longer in the transgenic plants than in the wild-type (Figure 7A,B). To confirm that these phenotypes were directly linked to altered flavonol contents, we performed in situ flavonoid staining of transgenic roots with diphenylboric acid 2-aminoethyl ester (DPBA), a fluorescent dye that specifically interacts with flavonols. Both wild-type and transgenic tobacco roots showed the specific fluorescence of the quercetin-DPBA complex (Q-DPBA), but fluorescence of the kaempferol-DPBA complex was not shown, indicating that the flavonol that mainly accumulates in the roots of tobacco seedlings is quercetin rather than kaempferol. The intensity of Q-DPBA fluorescence was stronger in transgenic vs. wild-type roots. These findings suggest that the increased quercetin levels in tobacco roots stimulate primary root and root hair growth. 

### 2.6. Exogenous Rutin Treatment Enhances Primary Root and Root Hair Growth in Wild-Type Tobacco Seedlings

**Figure 8:**
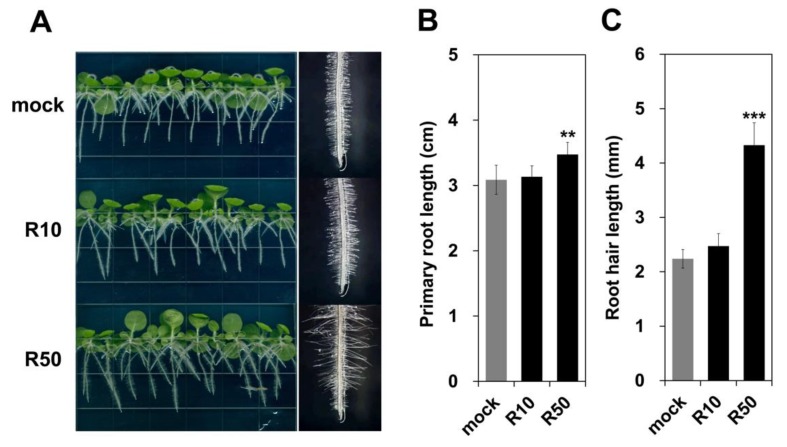
Root growth-promoting effects of rutin on tobacco seedlings. (**A**). 16-day-old wild-type seedlings grown vertically on MS medium (without sucrose) containing 10 µM (R10), 50 µM (R50) rutin, or DMSO (mock) were compared (left column). The root hair was observed by microscopic examination (right column). (**B**), Average primary root length of 30 seedlings. (**C**), Average root hair length of at least eight primary roots. Data are expressed as mean ± SD, as determined from the replicates. Significant differences were determined by Student’s *t*-test. Asterisks indicate that the value is significantly different from that of mock treatment (** *p* < 0.01, *** *p* < 0.001).

DPBA staining revealed only quercetin signals in the roots of tobacco seedlings. The corresponding compound was regarded as rutin, since kaempferol 3-*O*-rutinoside and quercetin 3-*O*-rutinoside (rutin) are the major flavonols that accumulate in tobacco [50]. Therefore, we grew wild-type seedlings vertically on Murashige and Skoog (MS) medium containing 10 or 50 µM rutin to verify the root growth-promoting effects of this compound. Seedlings grown on 10 µM rutin (R10) showed no significant differences in root growth compared to those grown under mock treatment, whereas treatment with 50 µM rutin (R50) significantly increased primary root and root hair length (Figure 8A). Primary root and root hair length in R50 increased approximately 12.6% and 93.4%, respectively, compared to mock treatment (Figure 8B,C). These results suggest that rutin is an important factor that positively affects root growth, particularly root hair growth, in tobacco seedlings. 

## 3. Discussion

Here, we investigated whether AcFLS-HRB is a bifunctional enzyme with both F3H and FLS activity. An in vivo substrate-feeding assay showed that flavanones could be converted to dihydroflavonols via the activity of AcFLS-HRB (Figure 2), indicating that AcFLS-HRB exhibits F3H as well as FLS activity. Among the FLSs that were enzymatically characterized in various plant species, most FLSs (OsFLS, AtFLS, CitFLS, GbFLSn, and AcFLS) are bifunctional enzymes, except for ZmFLS1 [30], suggesting that FLSs are able to substitute for the role of F3H in flavonoid biosynthesis in many plant species. Indeed, flavonols and anthocyanins are produced in the *tt6* (*F3H* deficient) mutant, but at much lower levels than in the wild-type [51,52]. Interestingly, unlike the wild-type, *tt6* seed coats are yellow/green at harvest but turn pale brown over time [51]. Thus, it appears that the F3H activity of FLS can augment or substitute for the function of authentic F3H enzyme, but dihydroflavonol biosynthesis by FLS is controlled in a manner different from that of F3H.

We successfully expressed the monocotyledonous *AcFLS-HRB* gene in dicotyledonous tobacco, which increased flavonol levels and decreased anthocyanin levels in tobacco flowers (Figure 3 and Figure 4). This confirmed that *AcFLS-HRB* is a functional *FLS* gene in planta. *ZmFLS1* and *OsFLS*, other monocotyledonous *FLS* genes that were functionally characterized in planta, successfully produced flavonol in *Arabidopsis* and tobacco, respectively [30,50]. These findings indicate that the functionality of *FLS* is well conserved between monocot and dicot plants. In the current study, the *AcFLS-HRB* transgene was highly expressed in tobacco petals; however, the total flavonol levels in the petals increased by only 5% to 28% compared to WT petals. These results appear to be due, in part, to the failure of AcFLS-HRB to interact with the flavonoid biosynthetic enzymes in tobacco. To optimize the catalytic efficiency of enzymes and co-ordinate metabolic crosstalk, sequential enzymes interact with each other to form supramolecular complexes called metabolons [53]. There is mounting evidence that flavonoid biosynthesis enzymes are able to form a metabolon, with the membrane-bound P450 enzyme F3ʹH functioning as a scaffold to bind to soluble enzymes during metabolon formation [53,54]. In the current study, most of the AcFLS-HRB protein was detected in the soluble fraction, with trace levels detected in the insoluble microsomal fraction (Figure 5B), suggesting that the number of metabolons may be insufficient to accommodate a large number of AcFLS-HRB, which may limit the metabolic capacity for flavonoid biosynthesis. Alternatively, the interaction between AcFLS-HRB and the endogenous tobacco enzymes may not be efficient due to a lack of compatibility. For these reasons, the flavonols do not appear to accumulate above a certain threshold, even in plants overexpressing *FLS*. Similarly, an *Arabidopsis fls1* mutant complemented by *ZmFLS1* accumulated flavonols at levels slightly lower than those of wild-type *Arabidopsis* [30]. Likewise, the transgenic expression of *FLS* genes isolated from rose, peach, or petunia in tobacco resulted in a 35% increase in flavonol levels, on average [24]. However, another study showed relatively high levels of flavonol accumulation: the expression of *Brassica napus FLS* (*BnFLS*) in *Arabidopsis* resulted in an 85% increase in flavonol levels relative to the WT [55]. Considering that *Brassica napus* and *Arabidopsis* both belong to the Brassicaceae family, the relatively high levels of flavonol in the transgenic plants may be due to high compatibility between BnFLS and the flavonoid biosynthetic enzymes in *Arabidopsis*. 

Our expression analysis showed that genes in the early and late steps of the flavonoid biosynthesis pathway were suppressed in transgenic petals (Figure 6). In the case of *FLS* suppression, a decrease in flavonols and an increase in anthocyanin levels typically occur in flower petals [20,21,22], but the expression of the other flavonoid biosynthetic genes are not affected [22], suggesting that the lack or reduced levels of flavonols do not affect the regulation of the flavonoid biosynthesis genes, and the metabolic flux simply flows to DFR in the absence of FLS. On the other hand, excessive flavonol levels due to *FLS* overexpression, as in our case, may cause the down-regulation of EBGs and LBGs in flower petals, indicating that flavonols can be causal compounds in the negative regulation of flavonoid biosynthesis. The branch point separating the flavonol and anthocyanin biosynthesis pathways is an attractive target for the molecular breeding of flower color. To obtain more intense flower color, suppression of *FLS* and overexpression of *DFR* were applied simultaneously [21,56]. However, for a stronger effect, it is necessary to increase the overall metabolic flux in this pathway. For this, it could be effective to use appropriate transcription factors. In addition, if a flower-specific promoter is used, the desired plant could be successfully obtained without affecting plant growth [57]. However, when flavonol levels are excessively reduced by *FLS* suppression, seed set could be arrested, because flavonols are involved in auxin transport [58]. Thus, this aspect should be taken into account when designing strategies for obtaining enhanced flower color. 

In addition to changes in flower color, we observed increased root growth in *AcFLS-HRB*-overexpressing tobacco, suggesting that the increased flavonol levels in tobacco roots promote root growth. DPBA staining revealed only quercetin signals in transgenic roots, which were likely derived from rutin, since the major flavonols in tobacco are kaempferol 3-*O*-rutinoside and rutin [50]. Thus, we treated wild-type tobacco plants with rutin and verified its positive effects on root growth (Figure 8). The rutin levels in tobacco increased in response to *NtCHS* overexpression and decreased by *NtCHS RNAi* [59]. However, changes in primary root length were not observed in the transgenic plants compared to wild-type. The enhanced rutin levels increased salt tolerance in *NtCHS-*overexpressing tobacco plants, thus alleviating the decrease in root length caused by NaCl treatment. Plants treated with rutin plus NaCl had longer primary roots than plants treated with NaCl alone. Similarly, our results indicate that rutin promotes root growth in tobacco (Figure 8). However, flavonol accumulation or quercetin aglycone treatment suppresses root growth in *Arabidopsis* [36,60,61], pointing to differences in the regulation of flavonol-mediated root growth in tobacco vs. *Arabidopsis*. Interestingly, we detected significant increases in root hair length in transgenic tobacco and in rutin-treated wild-type plants, but the number of root hairs was not altered in both cases. The tomato *are* mutant, which contains trace level of flavonols but accumulates high levels of H_2_O_2_ in the root maturation zone, has more root hairs than the wild-type, but its root hair length is not altered [37], implying that due to the lack of flavonols, H_2_O_2_ scavenging does not occur, and thus root hair differentiation is highly activated. Therefore, perhaps the increased rutin levels in the root of *AcFLS-HRB*-overexpressing tobacco extensively scavenged H_2_O_2_ in the differentiation zone, which further arrested cell differentiation, resulting in the unaltered number of root hairs. Previously, it was observed that increased levels of rutin in tobacco more efficiently removed H_2_O_2_ than O_2_^−^ [59], and another study showed that the inhibition of O_2_^−^ bursts by blocking NADPH oxidase activity suppressed root hair elongation in *Arabidopsis* [62]. Therefore, we can hypothesize that high levels of rutin in tobacco root mainly scavenge H_2_O_2_ in the differentiation zone, O_2_^−^ would antagonistically replace it, and the consequent increase in the ratio of O_2_^−^ to H_2_O_2_ in this region may account for the promotion of root hair elongation. 

Different flavonol species and specific derivatives of flavonols (rather than aglycones) have different effects on PAT and ROS scavenging [39,41]. Given the unique intracellular compositions of kaempferol and quercetin and their derivatives in different plant tissues and species with distinct flavonoid modification systems, the regulation of flavonol-mediated root growth cannot be generalized to other plant species. Here, we showed that bifunctional AcFLS expression in tobacco resulted in changes in flower color and promoted root growth by enhancing flavonol levels. Our findings also suggest that rutin is a regulatory compound that promotes root growth.

## 4. Materials and Methods 

### 4.1. Expression of Recombinant AcFLS-HRB Protein in E. coli and In Vivo Feeding Assay

The *AcFLS-HRB* ORF was amplified using a set of specific primers (Table 1). The PCR products were cloned into the pGEX-4T-3 vector that had been linearized by *Bam*HI digestion using an InFusion Advantage PCR Cloning Kit (Clontech, Mountain View, CA, USA) in-frame with the sequence encoding the N-terminal glutathione *S*-transferase (GST) tag. The resulting vector, pGEX-4T-3-*AcFLS*, was verified by sequencing and transformed into *E. coli* strain BL21 (DE3) cells (Novagen, Darmstadt, Germany). Transformed bacterial cells were cultured in 50 mL of LB broth, and protein expression was induced by adding 0.1 mM isopropyl β-D-1-thiogalactopyranoside (IPTG) and incubation at 28 °C for 2 h. The racemic flavanone substrates naringenin and eriodictyol were simultaneously added to 2 mL of the induced culture to a final concentration of 100 μM of each compound. After 3 h of incubation at 28 °C, 800 μL of the culture was harvested, sonicated, and extracted with 1 volume of ethyl acetate. The ethyl acetate extracts were evaporated with nitrogen gas, and the residues were dissolved in 100 μL of methanol for HPLC analysis. Aliquots of bacterial cultures noninduced or induced by IPTG were subjected to SDS-PAGE to confirm the expression of recombinant GST-AcFLS-HRB protein.

### 4.2. Vector Construction for Tobacco Transformation

The *AcFLS* gene was isolated from the ‘Hwangryongball’ onion cultivar (AcFLS-HRB) and introduced into the pTOP Blunt V2 vector (Enzynomics, Daejeon, Republic of Korea) as previously described [49]. We carried out PCR using the resulting vector pTOP Blunt V2:*AcFLS-HRB* as a template, with a sequence-specific primer set (Table 1) designed to complement either end of the pE3c entry vector linearized by *Dra*I digestion. The PCR product was cloned into the *Dra*I-digested pE3c vector using an InFusion Advantage PCR Cloning Kit (Clontech), in-frame with the sequence encoding the C-terminal 6x c-myc tag. Subsequently, the coding region of *AcFLS-HRB* fused with the 6x c-myc tag was transferred to the destination vector pK2GW7 to generate the final pK2GW7-*AcFLS-HRB*-myc construct using the GATEWAY^TM^ system (Thermo Fisher Scientific, Waltham, MA, USA), according to the manufacturer’s instructions [63]. The final vector also contained the CaMV 35S promoter to drive the expression of the transgene and the kanamycin resistance gene for the selection of transgenic plants. 

### 4.3. Transformation of Tobacco Plants

Transgenic tobacco was generated by transforming leaf discs with *Agrobacterium tumefaciens* GV3101 containing the pK2GW7-*AcFLS-HRB*-myc construct as described previously [64]. Briefly, tobacco seeds were surface sterilized with 70% ethanol followed by 25% commercial bleach and grown on solidified half-strength Murashige and Skoog (MS) medium under a 16 h light/8 h dark cycle at 26 ± 1 °C for two months. Leaf discs were dissected and submerged in the *Agrobacterium* mixture. The explants were cultured on a shoot-inducing medium containing 50 mg·L^–1^ kanamycin to select transgenic calli. The regenerated shoots were subsequently transferred onto root-inducing MS medium to enable rooting prior to being transplanted into a greenhouse. The transgenic tobacco plants were grown to maturity under the same conditions as above and we obtained more than 20 T_0_ transgenic lines. Five or more T_1_ transgenic lines were generated from each self-pollination of the T_0_ lines. The flowers of three or more individuals in each T_1_ line showed decreases in pinkness to various extents compared to WT flowers. We classified the T_1_ lines into two groups (LP and PP) based on the observed changes in petal color and selected two representative plants from each group. Petals were collected from at least ten flowers of each representative plant and ground to a powder using liquid nitrogen for further analysis.

### 4.4. HPLC Analysis of Flavonoids

The accumulation of flavonoid aglycones in the petals was analyzed as described previously [57], with some modifications. Briefly, 100 mg of ground petal sample was subjected to a 2-h acid hydrolysis in 300 μL of 50% methanol containing 2 N HCl at 90°C. After centrifugation at 15,000× *g* for 10 min at 4 °C, the supernatant was transferred to a new tube. The remaining pellet was rinsed with 200 μL acid hydrolysis solution, which was then combined with the first extract. A 10 μL aliquot of the extract was used in a HPLC analysis performed on an LC-20A HPLC system (Shimadzu, Kyoto, Japan) equipped with an Inertsil-ODS3 C18 column (5 μm, 250 × 4.6 mm; GL Science, Eindhoven, Netherlands). The chromatographic separation was carried out using 0.1% formic acid in water (solution A) and 0.1% formic acid in acetonitrile (solution B) with the following gradient conditions; 0–30 min, linear gradient of 5–55% (*v*/*v*) solution B; 30-45 min, linear gradient of 55–65% (*v*/*v*) solution B; and 45–50 min, linear gradient of 65–100% (*v*/*v*) solution B at a flow rate of 1 mL∙min^−1^. The temperature of the column was maintained at 40 °C. A diode-array detector was used for compound detection. The spectra of the compounds were recorded between 210 and 800 nm, and the peak corresponding to each compound was identified by comparing the retention times and UV spectra with those of the standards.

### 4.5. Protein Extraction and Immunoblot Analysis

Each ground petal sample was mixed with three volumes of pre-chilled protein extraction buffer containing 50 mM Tris-Cl (pH 8.0), 2 mM EDTA, 250 mM sucrose, 2 mM DTT, and 200 μM phenylmethylsulfonyl fluoride. The extracts were collected in 1.5 mL tubes and centrifuged at 6000× *g* for 10 min at 4 °C, after which the supernatants were transferred to new 1.5 mL tubes. This fraction was referred to as the total protein. The soluble and microsomal proteins were separated from the total protein using a previously described method [65], with some modifications. NaCl and PEG4000 were added to the total protein solution to concentrations of 150 mM and 0.1 g·mL^−1^, respectively. After incubation on ice for 15 min, the microsomal fractions were collected by centrifugation (10 min, 10,000× *g*, 4 °C). The supernatants were transferred to new 1.5 mL tubes, and were referred to as the soluble protein. The collected microsomal pellet was resuspended in TEG buffer (50 mM Tris (pH 7.5), 1 mM EDTA, and 30% glycerol). Aliquots of the microsomal fractions were shock-frozen in liquid nitrogen and stored at −80 °C until further use. The protein concentrations were determined using the Bradford reagent (Bio-Rad Laboratories), and 12 μg of each protein sample was subjected to immunoblot analysis with the anti-c-myc antibody, as previously described [65].

### 4.6. RNA Extraction and qPCR Analysis

Total RNA was prepared from the petals of WT and transgenic tobacco flowers and the petals of mock- and flavonol-treated flowers using a FavorPrep™ Plant Total RNA Mini Kit (Favorgen, Pingtung, Taiwan), according to the manufacturer’s instructions. The cDNAs were synthesized from 1.5 μg total RNA using an amfiRivert cDNA Synthesis Platinum Master Mix (GenDEPOT, TX, USA). The qPCRs were performed using AccuPower 2x Greenstar qPCR Master Mix (Bioneer, Daejun, Republic of Korea) and the Bio-Rad CFX96 Detection System (Bio-Rad Laboratories, CA, USA), according to the manufacturer’s instructions. The expression levels of the target genes were normalized against the expression of the tobacco glyceraldehyde 3-phosphate dehydrogenase gene (NtGAPDH). The gene-specific primers used for qPCR analysis are listed in Table 1.

### 4.7. Tobacco Seedling Culture and Exogenous Rutin Treatment

Seeds of wild-type and transgenic (T_3_) tobacco were surface sterilized and grown on MS medium containing 0.4% plant agar without sucrose under a 16 h light/8 h dark cycle at 26 ± 1 °C for 8 days. Eight-day-old seedlings at the same growth stage were transferred to MS medium solidified with 0.8% gelrite in square dishes and grown vertically for eight more days. For exogenous rutin treatment, stock solutions were prepared by dissolving rutin in dimethyl sulfoxide (DMSO) at concentrations of 50 mM and 10 mM, which were added to the MS medium at a ratio of 1/1000 (*v*/*v*). For the mock treatment, only DMSO was added to the MS medium at the same ratio. Eight-day-old wild-type seedlings were transferred to these media and cultured vertically for eight more days. 16-day-old-seedlings were photographed on the dishes, and their root hairs were identified using a microscope. The primary root lengths of the 30 seedling were measured and averaged. We also measured the lengths of the longest root hair in the region up to 10 mm above the root tip in at least eight primary roots and then averaged.

### 4.8. DPBA Staining 

The roots of 16-day-old seedlings were cut, submerged in an aqueous solution of 0.25% (*w*/*v*) DPBA (Sigma-Aldrich, Steinheim, Germany) and 0.2% (*v*/*v*) Triton X-100, and incubated on a rotary shaker at 20 rpm for 2 h. The roots were washed three times with deionized water, and the fluorescence of flavonol-DPBA was visualized in vivo by confocal fluorescence microscopy (Leica TCS SP8; Leica Microsystems, Wetzlar, Germany). Fluorescence emission was captured at 475–504 nm for kaempferol and 577–619 nm for quercetin after excitation at 450–490 nm [66]. 

### 4.9. Chemical Standards

(±)-Naringenin, (±)-eriodictyol, (±)-DHK, (±)-DHQ, kaempferol, quercetin, and rutin hydrate were purchased from Sigma-Aldrich (St. Louis, USA), and cyanidin chloride and kaempferol-3-*O*-rutinoside were purchased from Extrasynthese (Extrasynthese, Genay Cedex, France). The flavanones, dihydroflavonols and flavonols were prepared as 100-mM stock solutions in DMSO, and cyanidin chloride was prepared to 100 mM in 50% methanol containing 1.2 N HCl. An external UV standard calibration was carried out to obtain the calibration curves for kaempferol, quercetin, and cyanidin chloride, which were used as standards to quantify the flavonol aglycones and glucosides, and cyanidin aglycones and glucosides. 

### 4.10. Statistical Analysis

Data were expressed as the mean of three independent replicates. Statistical significance was evaluated by one-way ANOVA with post-hoc Tukey’s tests using the SPSS Statistics program (version 25, IBM, NY, USA).

## Figures and Tables

**Table 1:** List of primers used for cloning and qPCR.

Target	Forward (5′→3′)	Reverse (5′→3′)	Usage
*AcFLS*	AAAAAAGCAGGCTTTATGGAAGTAGAGAGA	TGAATTGGTTCCTTTCCCTGAGGAAGTTTATT	Cloning
*AcFLS*	ACACTGACATGTCCAGCCTCACC	TTACCGTTGTTCTGTGTAGCACGC	qPCR
*NtPAL*	ATTGAGGTCATCCGTTCTGC	ACCGTGTAACGCCTTGTTTC	qPCR
*Nt4CL*	TCATTGACGAGGATGACGAG	TGGGATGGTTGAGAAGAAGG	qPCR
*NtCHS*	TTGTTCGAGCTTGTCTCTGC	AGCCCAGGAACATCTTTGAG	qPCR
*NtCHI*	GTCAGGCCATTGAAAAGCTC	CTAATCGTCAATGCCCCAAC	qPCR
*NtF3H*	CAAGGCATGTGTGGATATGG	TGTGTCGTTTCAGTCCAAGG	qPCR
*NtF3′H*	AGGCTCAACACTTCTCGT	CATCAACTTTGGGCTTCT	qPCR
*NtFLS*	TTTGGCACTTGGTGTTGTGG	ACTTGACATCATACCAATGG	qPCR
*NtDFR*	AACCAACAGTCAGGGGAATG	TTGGACATCGACAGTTCCAG	qPCR
*NtANS*	TGGCGTTGAAGCTCATACTG	GGAATTAGGCACACACTTTGC	qPCR
*NtGAPDH*	GGTGTCCACAGACTTCGTGG	GACTCCTCACAGCAGCACCA	qPCR
